# Charge and spin transport in single and packed ruthenium-terpyridine molecular devices: Insight from first-principles calculations

**DOI:** 10.1038/srep31856

**Published:** 2016-08-23

**Authors:** C. Morari, L. Buimaga-Iarinca, I. Rungger, S. Sanvito, S. Melinte, G.-M. Rignanese

**Affiliations:** 1 National Institute for Research and Development of Isotopic and Molecular Technologies (NIRDIMT), 65-103 Donath, Ro-400293, Cluj-Napoca, Romania; 2 School of Physics and CRANN, Trinity College, Dublin 2, Ireland; 3 National Physical Laboratory, Teddington, TW11 0LW, United Kingdom; 4 ICTM Institute, Université catholique de Louvain, 1348 Louvain-la-Neuve, Belgium; 5 IMCN Institute, Université catholique de Louvain, 1348 Louvain-la-Neuve, Belgium; 6 European Theoretical Spectroscopy Facility (ETSF), 1348 Louvain-la-Neuve, Belgium.

## Abstract

Using first-principles calculations, we study the electronic and transport properties of rutheniumterpyridine molecules sandwiched between two Au(111) electrodes. We analyse both single and packed molecular devices, more amenable to scaling and realistic integration approaches. The devices display all together robust negative differential resistance features at low bias voltages. Remarkably, the electrical control of the spin transport in the studied systems implies a subtle distribution of the magnetisation density within the biased devices and highlights the key role of the Au(111) electrical contacts.

The study of metallic complexes has been a mainstay for molecular electronics^1^,^2^ and metallo-supramolecular chemistry^3^,^4^,^5^. Numerous experimental investigations, accompanied by theoretical approaches^6^, were conducted in the search for possible molecular building blocks for future devices with tunable magnetic behaviour^7^,^8^,^9^,^10^,^11^,^12^. Seminal device fabrication-driven searches have been directed to the study of ordered thin films, from multilayers to monolayer assemblies^13^,^14^,^15^,^16^,^17^,^18^,^19^,^20^, and addressed the behaviour of single molecules on metallic surfaces^21^,^22^,^23^,^24^,^25^,^26^.

Among the wide variety of candidates for single-molecule two-terminal devices^27^,^28^,^29^, compounds incorporating a single transition metal ion constitute a privileged category^30^,^31^,^32^,^33^,^34^. In particular, the presence of a transition metal ion allows the tuning of the electronic transport through well-defined charge and possibly spin states of the molecular species. Recent experimental work on charge transport in transition metal coordination complexes revealed a rich physics including inelastic tunneling effects, negative differential resistance and Kondo resonances^32^,^35^. The case for transition metal coordination complexes is thus twofold. From the experimental point of view, there remains a strong interest to carefully consolidate the salient features that have come off intriguingly from single-molecule electrical measurements, and which will likely be expanded in technologically relevant molecular devices. Equally important, several numerical methodologies developed in the last decade play a considerable part in embodying new physical concepts and in designing critical experiments in molecular electronics. Accordingly, a vivid theoretical effort is currently devoted to the understanding and prediction of the unique charge transport signatures of coordination complexes and metal-organic frameworks^36^,^37^,^38^,^39^,^40^,^41^,^42^. Here we study the 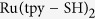 cardan-joint-like molecule, sandwiched between two Au(111) electrodes, where tpy-SH represents 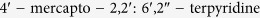. The Ru ion is bonded within an approximately octahedral environment to the two terpyridine derivatives linked to the Au(111) surfaces.

## Methods

The calculations are performed with SIESTA^43^,^44^ and SMEAGOL^45^,^46^,^47^ codes. We employ Troullier-Martins pseudopotentials^48^,^49^ and localized basis sets^43^. For the molecules we use a double zeta polarized basis set. For Au we use a single zeta for 5*d* orbitals and a double zeta polarized basis set for 6*s* orbitals. On the one hand, our choice is motivated by the computational effort; see below the description of the geometric models used in the calculations. On the other hand, this choice is indeed reasonable since the 5*d* orbitals of Au lay at about 2 eV below the Fermi level and therefore they do not take part in the transport. We note that alike basis sets were successfully used in previous transport calculations^50^,^51^. The energy shift^43^ used to define the Au orbitals is optimized by taking the geometry of the bulk Au as reference. We found that a 300 meV energy shift for Au leads to a computed bulk lattice parameter of 4.098 Å, close to the experimental value of 4.078 Å. For the S, Ru, N, C, and H atoms, the energy shifts are chosen employing as reference the geometry of the molecule optimized using a plane-wave method^52^. We inferred that the smallest forces (about 0.4 eV/Å) are obtained with a 25 meV energy shift.

For all the investigated geometric models, we use generalized gradient approximation^49^ functionals. We employ periodic boundary conditions in the direction orthogonal to the transport direction, sampling over a uniform 2 × 2 grid of *k*-points in the two-dimensional Brillouin zone. In the transport calculations, the Green’s function leading to the density matrix is integrated typically over 24 energy points on the complex semi-circle, 16 points along the line parallel to the real axis and 8 poles. The integral over real energies at finite bias is evaluated over about 180 points. The real-space grid cutoff is chosen to give an equivalent plane-wave energy cutoff of 250 Ry.

**Figure 1:**
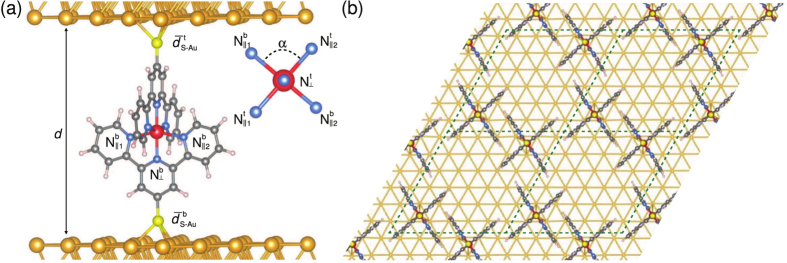
Ball and stick representation of the different models. (**a**) Side view of single molecule devices (*Models* 1–6 and 2B without Ru atom) and (**b**) top view of the packed molecular device (*Model* 2P). The Au, S, Ru, N, C, and H atoms are in gold, yellow, red, blue, gray, and white. For *Model* 2P, a 2 × 2 unit cell (dotted lines) is represented. For the sake of clarity, only the bottom electrode is shown and it is trimmed to the first atomic layer accommodating the linking S atoms in hollow positions.

In all our models, the electrodes consist of five Au(111) layers. For single molecule devices, we adopt a 5 × 5 surface cell resulting in a lattice constant of 20.49 Å based on the optimized lattice constant of bulk Au. When attaching a molecule to the electrodes, the most stable configuration is achieved when the S atom is located at the hollow position with respect to the Au(111) surface, with a S-Au distance of ~1.8 Å. Therefore, this hollow position is adopted in all models. However, four different distances between electrodes [*d* in Fig. 1(a)] are considered, leading to S-Au bond lengths^53^ in line with typical experimental conditions. The corresponding models are referred to as *Model* 1, *Model* 2, *Model* 3 and *Model* 4, with supercell dimensions in the direction perpendicular to the surface of 37.68, 37.98, 38.28, and 38.48 Å.

In order to obtain a better sampling of the geometric configurations of the molecule linked to Au(111) electrodes, different dihedral angles [*α* in Fig. 1(a)] for the bridging ruthenium ligand are also considered. The investigation of alike molecular deformations is justified by the fact that experimental investigations are usually carried by inserting the dithiol-tethered ruthenium-bis(terpyridine) complex into an alkanethiol self-assembled monolayer^23^ or via mechanically breakable junctions^25^. We thus take into account two bent conformations with respect to the relaxed structure of the isolated molecule [Fig. 1(a) and Supplementary Information], while keeping the distance between electrodes identical to that used in *Model* 2. These latter models will be labeled as *Models* 5 and 6, respectively. Furthermore, in order to better understand the role of the Ru atom in the electronic transport, we also consider *Model* 2B which is obtained by removing the Ru atom from *Model* 2. It thus consists of two bare terpyridine units attached to the Au(111) electrodes and it is specified below as Au-S-tpy − tpy-S-Au.

The mono-molecular devices discussed until now have obvious advantages when considering the fine tuning of the interelectrode distances; however, their performance is seriously restricted to the capability of controlling precisely their molecular geometry. Robust negative differential resistance^54^,^55^ devices are sought after for technology purposes, and monolayers represent the next step to improve upon the performance of single-molecule devices. Therefore, in order to prove the efficiency of the 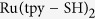 molecules on account of the presence of negative differential resistance behaviour, we studied a more complex two-electrode system (*Model* 2P) depicted in Fig. 1(b) which mimics the effects arising in self-assembled monolayers. The previously adopted 5 × 5 surface cell is too small to accommodate two molecules with a reasonable distance between them. Thus, *Model* 2P is built by adopting a 6 × 6 surface cell which allows for a minimum distance between the nearest atoms larger than 2.5 Å. The distance between the electrodes is chosen to be the same as in *Model* 2. The two molecules are arranged in a grid-like topology, inducing a porous two-dimensional network.

For all the models, the atomic positions of the molecules are relaxed together with the first two atomic layers of the Au(111) electrodes until the forces are smaller than 0.01 eV/Å, while keeping the dimension of the supercells constant and pinning the remaining atomic positions of the Au electrodes to their bulk positions. The relaxed structures can be characterized by the bonding of the molecules to the surface, the atomic displacements at the surface, and the molecular geometry deformation as reported in Table 1.

For *Models* 1 to 6, it is even possible to directly compare the different computed total energies. We found that *Model* 3 represents the most stable geometry of the molecule in contact with the semi-infinite Au(111) electrodes. Taking *Model* 3 as a reference, we first note that on pulling the electrodes apart (*Model* 4), as expected, the energy increases before the breaking of the molecular junction. Second, it can be observed that the reduction of the distance *d* between the electrodes induces a marked instability: a decrease of the distance between the two gold electrodes by 0.6 Å leads to an increase of the total energy by about 0.6 eV. In contrast, the variation of the dihedral angle *α* for the bridging Ru atom also leads to intriguing behaviour from the energetic point of view: *Models* 5 and 6 are more stable than *Model* 2. This is likely a consequence of the fact that the relative bending of the terpyridine units favours a dihedral angle *α* closer to 90° and allows thus the S-Au bonds to assume larger length values.

We also found slight differences for the two S atoms linked on the two metallic contacts in terms of the average bond length 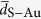. For each electrode, the differences among the lengths of the S-Au bonds for a given S atom were less than 0.02 Å in all cases, within the numerical accuracy of the method. The smallest differences between 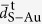 and 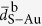 (where the t and b superscripts refer to the top and bottom electrodes, respectively) were observed for the most stable configuration (*Model* 3), while the largest differences were computed for *Models* 5 and 6 as well as for the control system provided by *Model* 2B.

The lengths of the 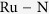 bonds are also reported in Table 1, distinguishing two values according to their relative orientation (|| and ⊥) with respect to the surface of the Au(111) electrodes, as depicted in Fig. 1(a). We observe that the values are in agreement with the previous studies^25^ and are essentially unchanged across the studied mono-molecular devices.

For *Model* 2P, the inter-electrode distance is *d* = 16.75 Å, similar to *Models* 2, 5, 6 and 2B. For each of the two molecules of the unit cell, the 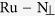 bond length is 2.00 Å and the 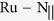 bond length is 2.09 Å. The dihedral angles are 86.71° (Molecule 1) and 86.78° (Molecule 2), respectively, for the two 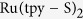 accommodated in the unit cell, smaller than those computed for the mono-molecular devices. Due to the packing, the molecules slightly adapt their distances with respect to the electrodes. One of them (Molecule 1) is slightly closer to the top electrode (unlike any of the previous models) with distances 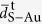 = 2.56 Å and 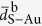 = 2.59 Å. In contrast, the other (Molecule 2) is closer to the bottom electrode with 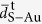 = 2.61 Å and 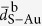 = 2.54 Å very similar to *Model* 2, 5 and 6. Moreover, this is the largest calculated difference between 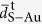 and 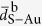.

For the SMEAGOL calculations^45^,^46^,^47^, the extended molecules consist of the geometric models mentioned above (Fig. 1) including only the first three electrode layers. There is no need to use more layers due to the short screening length in Au.

## Results

**Figure 2:**
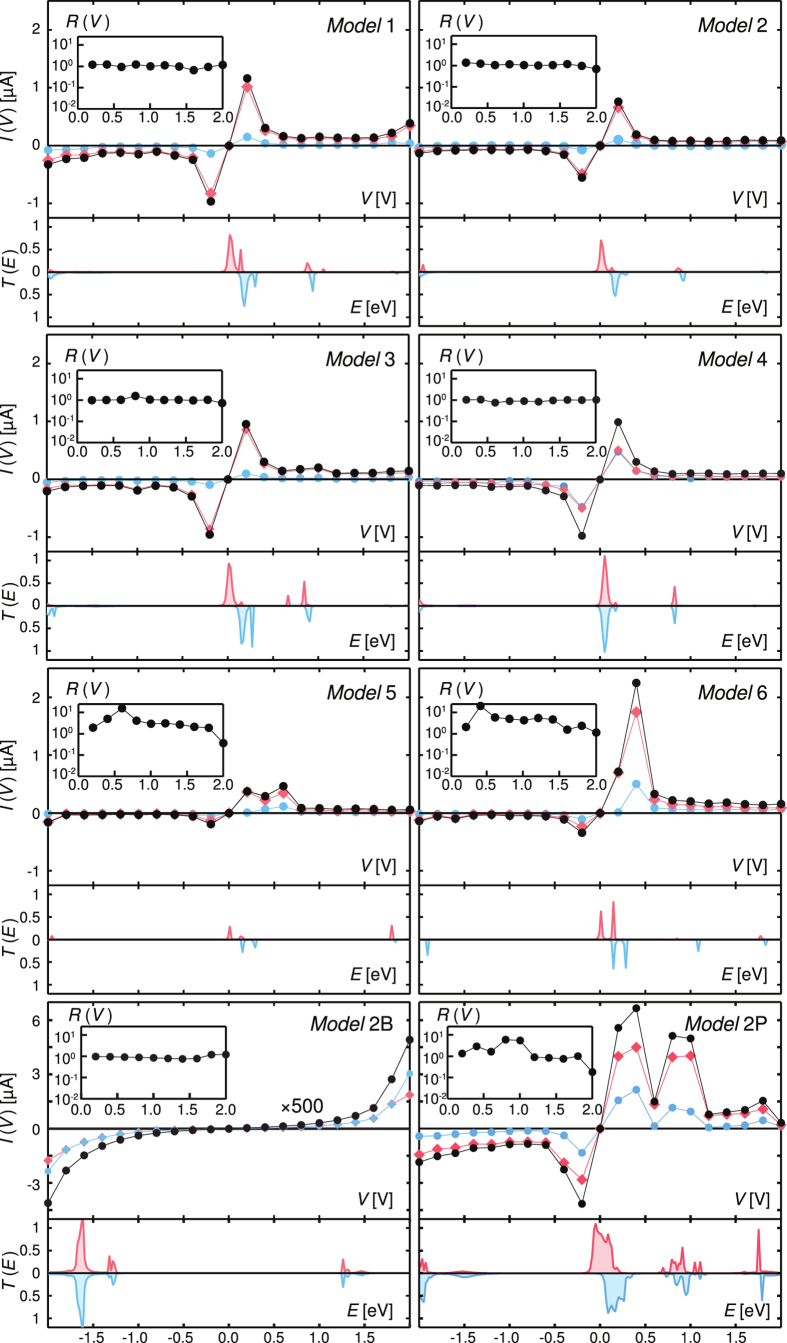
Transport properties of the different models. For all the models, the top panel shows the *I*(*V*) characteristics, while the bottom one displays the transmission *T*(*E*). The insets report the rectification ratio *R*(*V*). Red and blue curves and symbols refer to the spin-up and spin-down components, while black curves and symbols represent the total current. The Fermi level was set to zero. Note that, for the packed molecular model, the reported values have been divided by a factor of 2 for an easier comparison with the results obtained for single-molecule devices.

The computed transmission spectra at zero bias *T*(*E*) for all models are presented in Fig. 2 for the energy range from −2 to 2 eV around the Fermi level. For *Models* 1–6, the most interesting feature is the presence of two sharp peaks for the two spin channels, located near the Fermi level, around 0.1–0.2 eV. The existence of these two peaks can be explained as follows. Upon linking of the molecule to the metallic electrodes, the degeneracy of its frontier orbitals (see Supplementary Information) is lifted due to small structural distortions of the terpyridine ligands forming the cardan-joint-like molecule. This splitting is further eased by the proximity of these orbitals to the Fermi level and by the fact that the ligands are positioned differently from the top and bottom electrodes. A comparison with *Model* 2B, reveals that the presence of the bridging Ru atom has crucial consequences for the calculated transmission and the electrical characteristics of the Au-molecule-Au devices. Several differences between the studied cases can be identified by analysing the *T*(*E*) spectra. For instance, referring to the two maxima near the Fermi level, one of the peaks for *Models* 1–4 has a transmission close to unity, while the other displays a significantly reduced value, in contrast to the allure of the corresponding transmission peaks for *Models* 5 and 6. We note that for *Model* 5 the intensities of the spin-resolved transmission channels near the Fermi level are strongly diminished. Qualitatively, a dramatic suppression of the transmission and electrical conductance is indeed expected for a molecule with a dihedral angle *α* of 90° between the two rigid terpyridine ligands.

While the full width at the half maximum of the transmission peaks located close to the Fermi level also differs significantly among the various studied models and strongly suggests the asymmetry of the interactions of the molecule with the two Au(111) electrodes, the *T*(*E*) data equally displays intriguing peculiarities for *Models* 1–6 around 1 eV and in the *E* ≥ 1.5 eV energy range. For *Models* 1–4, relatively strong *T*(*E*) peaks at *E* ≤ 1 eV are accompanying those close to the Fermi energy, in contrast to *Models* 5 and 6 where the transmission is negligible in the corresponding energy range. In order to investigate the origin of the different *T*(*E*) peaks, the Ru, S, C and N projected density of states (PDOS) was computed for all cases (see Supplementary Information). The most important contributions are those of Ru and N atoms for all molecular levels participating in the build-up of the *T*(*E*) spectra, including the orbitals adjacent to the Fermi level.

For *Model* 2P, the calculated transmission spectrum encompasses the contributions of the two molecules, displaying an enhanced mixing of the frontier orbitals and recalling thus the well-known metal-metal coupling mediated by a bridging ligand. The three transmission peaks at about 0.25, 0.75 and 1.75 eV are well spread and display high intensities. Similar to previously discussed molecular models, we attribute these spectral features to hybridised frontier orbitals with enlarged spatial extension, lying above the Fermi level. These broad peaks are in register with marked PDOS features for the Ru and N atoms (Supplementary Information).

The current-voltage *I*(*V*) characteristics for all models are reported in Fig. 2 for biases between −2 to 2 V. For *Model* 2B, as expected, the transport occurs in the tunnelling regime, with a non-linear *I*(*V*) behaviour appearing at high bias without the presence of any resonance phenomena. For the *Models* 1–6, the *I*(*V*) characteristics typically present negative differential resistance (NDR) features with maxima located at *V* = ±0.2 and ±1 V, in close agreement with the intensity of the peaks displayed by the *T*(*E*) spectra. The investigations which have been carried out so far on the NDR in molecular systems included several classes of local probes and nano-patterned electrodes^56^,^57^,^58^. The NDR has been observed in various molecules accommodating metal atoms^59^,^60^,^61^ and different molecule-electrode interfaces^62^,^63^,^64^,^65^. Early attempts to construct devices exhibiting NDR recognised the essential role of the surrounding medium and electrodes^61^,^62^,^66^,^67^,^68^,^69^. The appearance of nearly symmetric NDR features in molecular junctions can be related to various mechanisms including molecular motion and electron-vibron coupling^70^, as well as complex spatial profiles of the electrostatic potential and variable tunnelling couplings of electrodes to the investigated specimens^26^. In the present case, the molecular charging of the frontier orbitals and their spatial configuration localised on the cardan-joint-like linked terpyridine ligands are playing an essential role for the observed NDR features, in conjunction with the tpy-S-Au orbital overlaps. Indeed, for *Models* 1–6, the NDR features originate from the orbital re-hybridization which appears under the applied bias and that dynamically changes the effective coupling between the molecule and the leads^71^. We note that for *Models* 1–4 nearly symmetric current-voltage curves are computed, and the presence of the second current peak at *V* = ±1 V is most discernible for *Model* 3. Interestingly, the *V* = 1 V peak weakens for *Model* 4, *i*.*e*. upon stretching the molecular junction. The use of Au(111) electrodes with variable inter-distances induces thus structural changes in the bridged molecule as registered by the slightly different shapes of the *I*(*V*) curves. By comparing the values of the total current at positive and negative voltage values for *Models* 1–4, we observe small discrepancies, which are attributed to the non-orthogonality of the two terpyridine units and the differences of the average S-Au bond lengths for the two metallic contacts. The overall asymmetry between the positive and negative biases is enhanced for *Models* 5 and 6. Indeed, for *Models* 5 and 6 the *V* = 0.2 V peak broadens significantly compared to *Model* 2 and its maximum seems dictated by the conformation of the molecule. For *Model* 5, since the twisted terpyridine units are adopting a perpendicular conformation leading to an almost total electronic decoupling of the two ligands, the shape of the *I*(*V*) curve becomes similar to that of *Model* 2B. In contrast with the present results, when molecules are inserted in self-assembled alkanethiol monolayers^23^, the cardan-joint effect is reduced; thus, this type of devices is mostly prone to redox-reaction-induced switching occurring at higher voltages. For *Model* 2P, the tuning of the molecular energy levels by biasing the metallic electrodes gives rise to a *I*(*V*) characteristic with pronounced wide resonances at the transmission peaks. A non-negligible excess electric current is found to flow for biases corresponding to energies within the *T*(*E*) forbidden gap. The monolayer device displays an intriguing multi-peak NDR behaviour: while the packed molecules still display the cardan-joint, symmetric NDR effect at low voltage, our calculations indicate that other mechanisms impact the *I*(*V*) curve as well. Indeed, the asymmetric peaks at positive bias voltages 1 and 1.7 V, the latter although of much weaker magnitude, resemble the well-known characteristics of resonant tunnelling diodes^54^,^55^. Such multi-peak NDR phenomena have been probed in electro-active molecules^72^ and are in agreement with both scenarios involving tunnelling through discrete unoccupied states of the specimens and measurement regimes controlling the redox centres of the molecules^23^.

**Figure 3:**
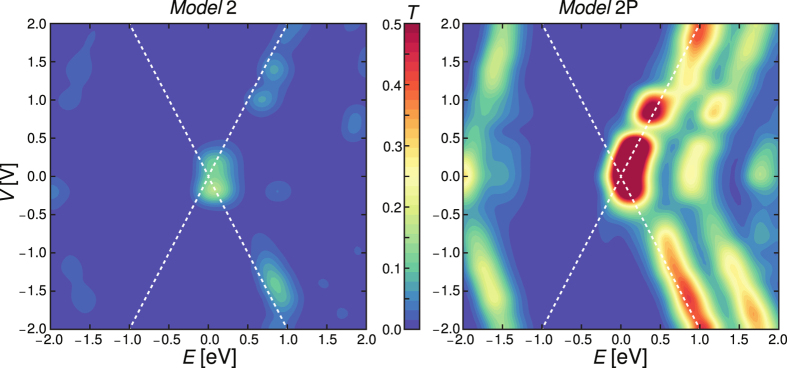
Contour maps of the total transmission *T* as a function of the energy *E* and the applied bias voltage *V* for *Models* 2 and 2P. The Fermi energy is set to zero on the energy axis. The white dotted lines indicate the chemical potential window as a function of the bias voltage *V*.

**Figure 4:**
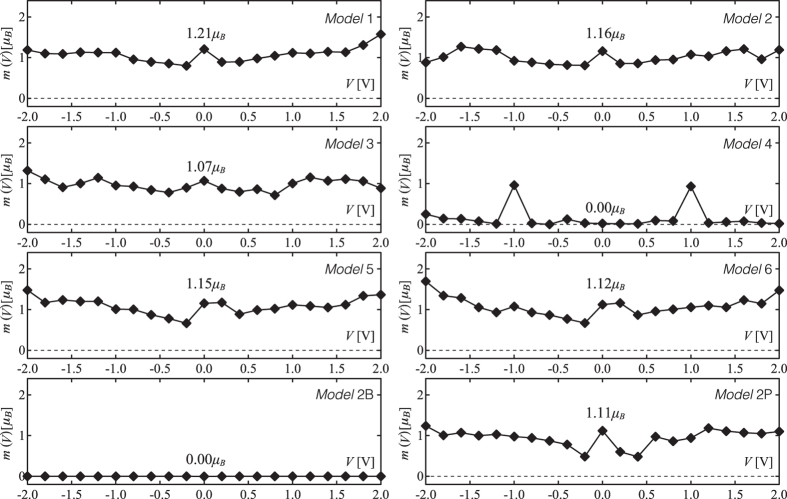
Calculated total magnetic moment *m* (in *μ*_*B*_) as a function of the applied bias *V* for the *Models* 1–6, 2B, and 2P.

**Figure 5:**
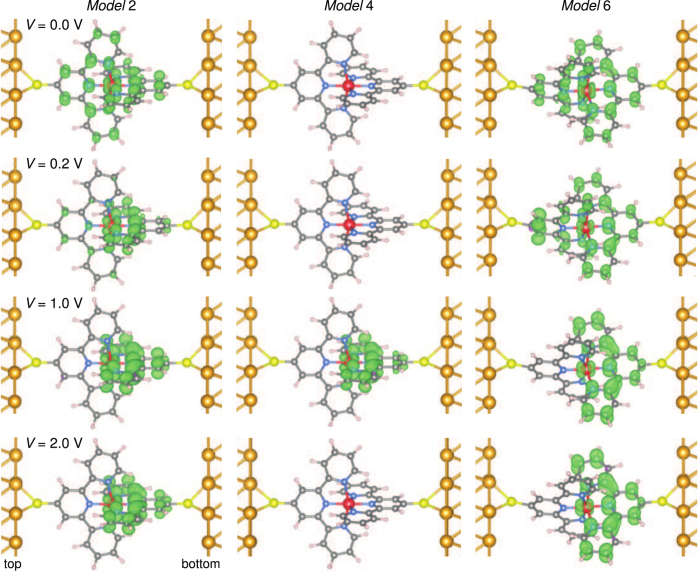
Magnetization density for *Models* 2, 4 and 6 at various voltages (*V* = 0.0, 0.2, 1.0 and 2.0 V). The atoms are represented adopting the same color scheme as in Fig. 1. The isosurfaces in green and violet correspond to a magnetization density of ±2 × 10^−3^ *μ*_*B*_/B*ohr*^3^.

The bias dependent total transmission as a function of energy is represented in Fig. 3 as contour maps for *Model* 2, representative for *Models* 1–6, and *Model* 2P. Our results for *Model* 2, at 1 and 2 V, are in line with those reported by Dhungana *et al*. (ref. 12, Fig. 3, bottom panels, ~1 and ~2 V) for similar devices. In both studies, the transmission peaks are displaced to higher energy values upon increasing the bias voltage. Furthermore, for both our *Models* 2 and 2P transmission peaks are also present in proximity of the Fermi level within the chemical potential window at low bias voltages, which translates into the appearance of NDR features. We do not expect a dramatic change in the position of these peaks (and hence NDR features) by taking into account more accurate correlation effects (Supplementary Information). Our findings are actually supported by experiments performed at low temperature on flexible alkyl-tailed cardan-joint specimens [ref. 73, Fig. 4 (panel B) and Fig. 5 (panel A)], which show the appearance of NDR features below 1 V (with stronger intensities as the temperature is decreased). Further details on the relation between the *T*(*E*, *V*) landscape and the appearance of NDR features in the transport characteristics can be found in the Supplementary Information.

The evolution of the spin-resolved and of the total currents is peculiarly interesting for the studied models, considering both the different distances between the Au(111) electrodes and the connections of the molecule in the two-electrode architectures. The Au(111) electrodes are non-magnetic. For the *Models* 2B and 4 the spin-up and spin-down components of the calculated electrical current are almost equal at low biases, suggesting that interfacial and symmetry-breaking, electrode-induced effects are likely not at the origin of the observed negative differential resistance in these devices. Upon shrinking the molecular junction, a highly spin-polarised electric current emerges at low bias for the considered cases: *Models* 1, 2, 3, 5, 6, and 2P. The main contribution to the total electric current arises from the spin-up channel, deriving from the frontier orbitals of the spin-filtering molecule and their preferential coupling with the electronic structure of the electrodes. The consequences on the spin-dependent transport caused by different values of the dihedral angle *α*, can be easily ascertained by comparing the spin polarisation curves 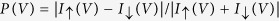 for *Models* 2, 5 and 6 (see Supplementary Information). For *Model* 2, an effective spin filtration of about 50% is seen at all biases and for both positive and negative bias voltages. However, for *Models* 5 and 6, a large spin polarisation of about 100% is seen at 0.2 V bias voltage and then it essentially reduces upon increasing the bias voltage. At negative bias voltages, the spin polarisation seems stable around 50%. Thus, the overall spin filtration seems dictated by the electrodes’ interdistance, while the dihedral angle and implicitly the average S-Au bond lengths are strongly influencing the behaviour at low bias voltages. For *Model* 2P, the overall spin filtration, as evidenced by the *P*(*V*) curve (Supplementary Information), is fully consistent to that observed in single-molecule devices.

We now turn to the molecular rectification effects displayed by the different investigated devices (Fig. 2). In agreement with the experimental protocols, we define the rectification ratio 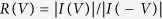 as the ratio of the total current amplitudes for opposite bias polarities. On the one hand, we note that for *Models* 1–4 rectification values close to unity are computed, since the *I*(*V*) curves are only slightly asymmetric and the rectifying character of the molecular devices is thus not enhanced. Obviously, practically no rectification is observed in the control *Model* 2B involving analogous symmetrical terpyridine ligands. On the other hand, it is quite interesting that for the nanojunction with the largest inter-electrode distance (*Model* 4), approaching the break junction disposition, unitary values for *R*(*V*) are observed at all biases. The rectification *R*(*V*) remains thus essentially unchanged when the molecule extends in the gap between the two Au(111) electrodes, while only upon very slight bending and torsion of the terpyridine ligands, calling for the re-adjustment of the S-Au bond lengths to match a given inter-electrode distance and to adapt towards a *α* = 90° conformation (*Models* 5 and 6), significantly higher values are obtained for *R*(*V*) at small biases. The *R*(*V*) curve of *Model* 2P also shows the robust features identified in the study of mono-molecular devices exemplified by the *Models* 2, 5, and 6. The monolayers are thus very effective in preserving the rectification ratios of about ~10 at small applied biases, where the values of the dihedral angle and of the S-Au bond lengths are pivotal. This highlights the ability of the bi-dimensional porous network of 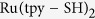 molecules to respond unequivocally to the applied external bias as exhibited by the *R*(*V*) curve.

## Discussion

The calculated values of the total magnetic moment *m* at different applied biases are summarized in Fig. 4 for all the studied molecular devices [Note that the self-energies of the metallic leads are included in the Hamiltonian]. At zero bias, a total magnetic moment is present for all cases with the exception of *Model* 2B and *Model* 4. Precisely, we obtain *m*(0) = 1.21, 1.16, 1.07, 1.15, 1.12, and 1.11 *μ*_*B*_ for *Models* 1–3, 5–6, and 2P (for the latter the value has been divided by two in order to take into account the presence of two molecules in the unit cell). It can be seen that *m*(0) increases when the electrode inter-distance decreases. In contrast, it is much less dependent on the dihedral angle and the packing as can be observed by comparing *Models* 2, 5, 6, and 2P. When considering the complete *m*(*V*) curve, it is less obvious to determine marked trends as a function of the distance, the dihedral angle, or the molecular packing (see superposition of the different curves in the Supplementary Information). However, on the one hand, *Model* 4 shows a clearly different behavior: the total magnetic moment is very small at all biases but *V* = ±1 V for which it shows a peak reaching *m* ~ 1 *μ*_*B*_. Interestingly, this is the same magnetization value as observed for *Models* 1–3 at the same bias. On the other hand, for *Models* 2, 5, 6, and 2P, the dependence on the dihedral angle and implicitly on the S-Au bond length is more pronounced in the low bias regime (for 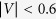 V).

In order to rationalise the differences in the transport and magnetic properties between the different topologies of the molecules connected to the two gold electrodes, the space-resolved spin densities for *Models* 2, 4 and 6 at various biases are given in Fig. 5. At *V* = 0 V, for *Models* 2 and 6 displaying a finite magnetic moment, the magnetic orbital is centered on the metal atom, yet delocalised on both ligands. Interestingly, for *V* = 0.2 V, the spin density retracts on the terpyridine linked to the bottom electrode for *Model* 2, while it spreads on both ligands for *Model* 6. At higher bias voltages, the magnetisation density is located on the bottom terpyridine for both *Models* 2 and 6. Note that this is the electrode where 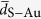 attains the smallest value. The same trend as a function of the applied bias was observed for all investigated molecular systems. Apart from the difference observed at low bias voltage, where the molecular conformation plays a major role, *Models* 5 and 6 behave similarly and in the same way as *Models* 1, 2 and 3. The magnetisation density plots for *Model* 2P at various biases are detailed in the Supplementary Information. For *Model* 4, where a magnetic moment is observed at 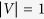 V, the space-resolved spin density overlaps the bottom terpyridine. The fact that the magnetic moment is not localised on a single atom (as detailed in the PDOS analysis displayed in the Supplementary Information) precludes the appearance of strong local correlations effects in the transport properties. The different distributions of the magnetisation density observed upon applying a small or a large bias, compared to that calculated at zero bias, could be exploited in the design of networks of molecular devices where the information is encoded by varying the magnetisation density throughout the array^73^,^74^.

## Conclusion

In summary, we have presented first-principles results for ruthenium-terpyridine molecules sandwiched between two Au(111) electrodes. Our analysis was performed using several geometrical models for the covalently coupled molecular-metal structures, including devices with single and packed molecules. The spin-resolved transmission spectra and the current-voltage characteristics were calculated for these two-terminal devices. Interestingly, for each investigated model, the presence of 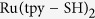 induces peculiar maxima in the equilibrium transmission function. In particular, their localization very close to the Fermi level leads to distinctive resonance peaks in the current-voltage curves at low bias. The computed electronic properties in the two-terminal devices are spin-dependent and the relative contribution of the two spin channels to the total electrical current and magnetic moment dramatically depends on the applied bias and the interelectrode distance. The studied systems open up new prospects for potential applications in molecular spintronic devices. For instance, the functionalization of the terpyridine units in the 4′ position with donor and acceptor groups, respectively, sitting thus on opposite directions one with respect to the other, could lead to molecular photodiodes with rigid geometries and unique spintronic properties at low temperatures.

## Additional Information

**How to cite this article**: Morari, C. *et al*. Charge and spin transport in single and packed ruthenium-terpyridine molecular devices: Insight from first-principles calculations. *Sci. Rep*. **6**, 31856; doi: 10.1038/srep31856 (2016).

## Supplementary Material

Supplementary Information

## Figures and Tables

**Table 1:** Structural properties of the different models: the inter-electrode distance *d*, the average S-Au bond length for the top (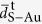) and bottom (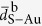) electrodes, the average Ru-N_⊥_ bond length, the average Ru-N_||_ bond length, the dihedral angle *α*, and the calculated energy with respect to the most stable model (that is *Model* 3).

*Model*	1	2	3	4	5	6	2B	2P	
*d* (Å)	16.45	16.75	17.05	17.25	16.75	16.75	16.75	16.75	
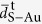 (Å)	2.54	2.60	2.64	2.69	2.60	2.62	2.58	2.56	2.61
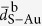 (Å)	2.52	2.56	2.63	2.67	2.57	2.57	2.54	2.59	2.54
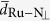 (Å)	2.00	2.00	2.01	2.01	2.00	2.00	1.98	2.00	2.00
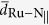 (Å)	2.08	2.08	2.09	2.09	2.08	2.08	2.31	2.09	2.09
*α* (°)	88.15	88.57	88.21	88.84	89.97	89.89	89.63	86.71	86.78
Δ*E* (eV)	0.60	0.20	0.00	0.07	0.04	0.04	‒	‒	‒

In the case of *Model* 2B (in which there is no Ru atom), the Ru-N distances correspond to half the N-N distances. The two columns for Model 2P correspond to the two molecules of the unit cell.
