# Supplementary material for: Charge and spin transport in single and packed ruthenium-terpyridine molecular devices: Insight from first-principles calculations
Source: Sci Rep. 2016 Aug 23;6:31856. doi: 10.1038/srep31856 (PMC4994010; doi:10.1038/srep31856)
Supplement: Supplementary Information [file srep31856-s1.pdf]

SUPPLEMENTARY INFORMATION

Charge and spin transport in single and packed ruthenium-terpyridine molecular devices : Insight from first-principles calculations

C. Morari,[†] L. Buimaga-Iarinca,[†] I. Rungger,[‡,‖] S. Sanvito,[‡] S. Melinte,[¶] and G.-M. Rignanese[*,§,⊥]

† *National Institute for Research and Development of Isotopic and Molecular Technologies (NIRDIMT), 65-103 Donath, Ro-400293, Cluj-Napoca, Romania*

‡ *School of Physics and CRANN, Trinity College, Dublin 2, Ireland*

¶ *ICTM Institute, Université catholique de Louvain, 1348 Louvain-la-Neuve, Belgium*

§ *IMCN Institute, Université catholique de Louvain, 1348 Louvain-la-Neuve, Belgium*

‖ *Materials Division, National Physical Laboratory, Teddington, TW11 0LW, United Kingdom*

⊥ *European Theoretical Spectroscopy Facility (ETSF)*

E-mail: gian-marco.rignanese@uclouvain.be

1 Methods

Our analysis was made using several geometrical models for the covalently coupled molecular-Au(111) structures, including devices with single and packed molecules. These investigated systems are labeled *Models* 1 - 6, 2B, and 2P, as described in the main text. For

the first-principles calculations we used the SIESTA and SMEAGOL codes. Electronic exchange and correlation were treated within the generalized gradient approximation. The two-dimensional Brillouin zone sampling was limited to a uniform 2 x 2 grid of *k*-points and the convergence threshold for the residual forces during atomic-structure relaxation was set to 0.01 eV/Å. The relaxed structures can be characterized by the bonding of the molecular units to the Au(111) surfaces and the molecular geometry deformation.[1] The atomic displacements at the electrode surface are presented below.

To quantify the distortion of the electrode surface in the studied systems, we computed the displacement of the Au atoms along the z-coordinate (*i.e.* the transport direction) from their averaged positions in the relaxed system:

$$\delta_z = \frac{1}{N}\sqrt{\sum_{i=1}^{N}(z_i - \langle z \rangle)^2}, \tag{1}$$

where N is the number of the atoms in the top layer of the surface, z_i is the current atomic position and $\langle z \rangle$ is the average value of the z-coordinate of the atoms in the top layer. For all models, the surface distortion is found to be negligible, with $\delta_z \approx 0.002$ Å, which is much smaller than the calculated distances $\bar{d}_{\mathrm{S-Au}}$, as summarized in Table S1.

Table S1: The S-Au bond lengths (in Å) of the investigated models for top and bottom electrodes.

Model	1	2	3	4	5	6	2B	2P	
								Molecule 1	Molecule 2
$\bar{d}^{t}_{\mathrm{S-Au}}$	2.54	2.60	2.64	2.69	2.60	2.62	2.58	2.56	2.61
$\bar{d}^{b}_{\mathrm{S-Au}}$	2.52	2.56	2.63	2.67	2.57	2.57	2.54	2.59	2.54

2 Computed electronic properties of the devices

For the interpretation of the transmission spectra $T(E)$ in the considered systems, related to the zero-bias conductance, we are referring to the projected density of states (PDOS) of the Ru, N, C and S atoms. The latter is computed using SIESTA on the whole system and applying a broadening of 0.1 eV. We represent the PDOS and the $T(E)$ results for *Models* 1 - 4 in Figure S1 and for *Models* 5, 6, 2B, and 2P in Figure S2.

2.1 Single and packed ruthenium-terpyridine molecules between two Au(111) electrodes: Negative differential resistance

The nonequilibrium transport method used for the SMEAGOL calculations is described in detail in Ref. 2. Basically, the current I flowing through the molecular junction is given by:

$$I = \frac{e}{h} \int dE \, T(E,V) \left[f(E-\mu_{\mathrm{L}}) - f(E-\mu_{\mathrm{R}}) \right], \tag{2}$$

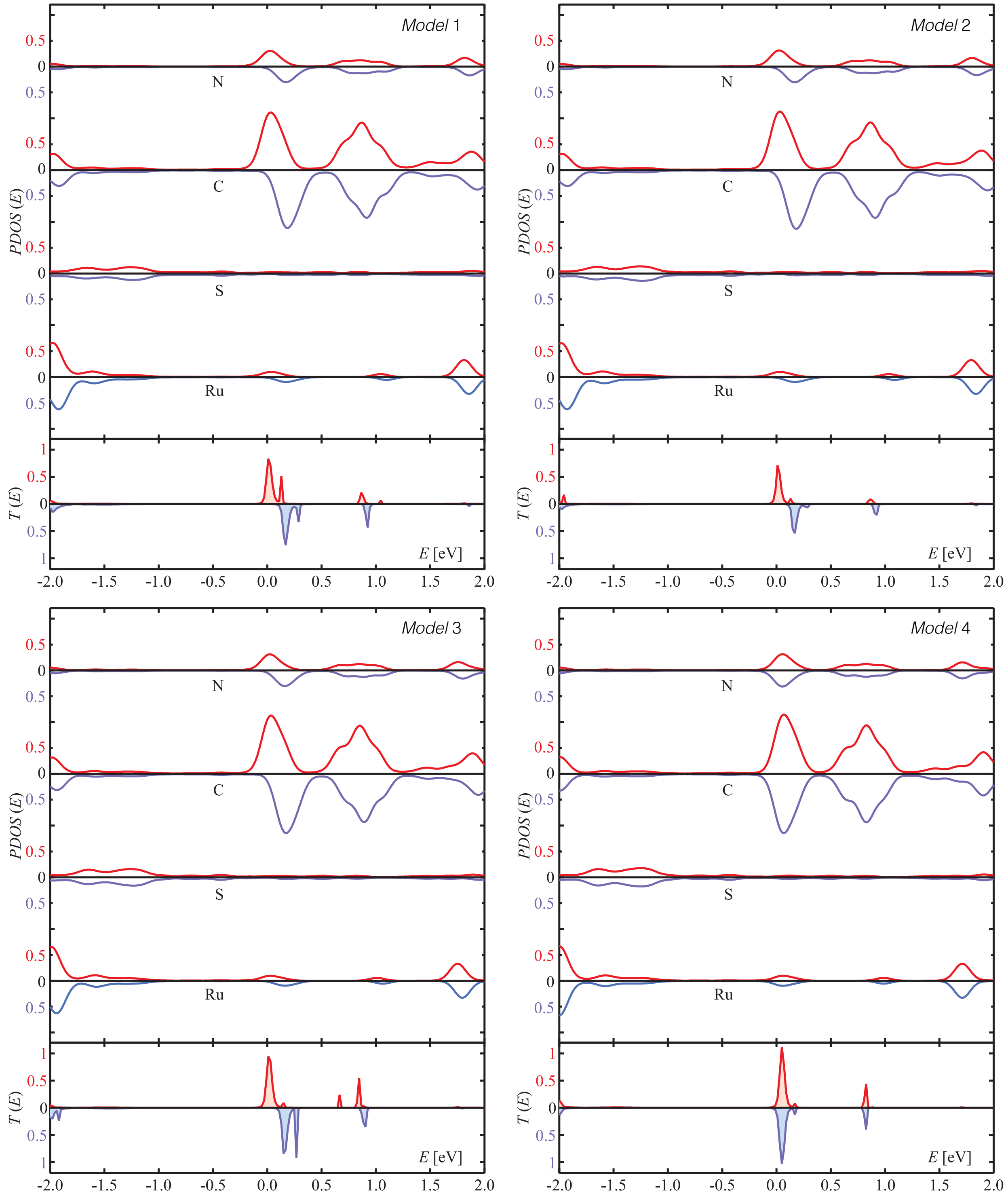

Figure S1: Transmission $T(E)$ and atom-projected density of states (PDOS) for *Models* 1 - 4. We use red curves for spin-up and blue curves for spin-down data. The Fermi level was set to zero. For each atom-projected density of states, the units correspond to 3 states/eV, while the transmission results are displayed between 0 and 1.

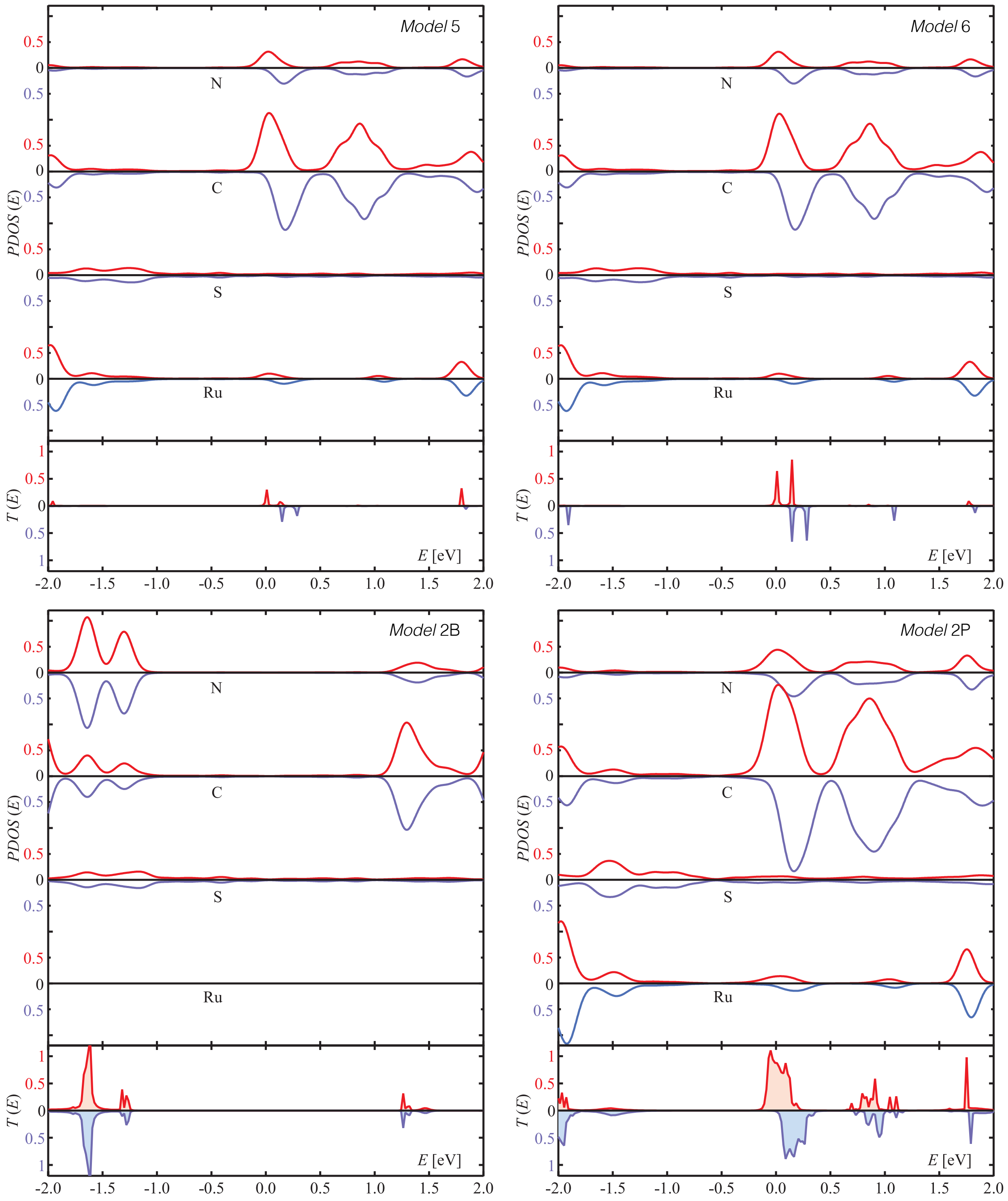

Figure S2: Transmission $T(E)$ and atom-projected density of states (PDOS) for *Models* 5, 6, 2B and 2P. We use red curves for spin-up and blue curves for spin-down data. The Fermi level was set to zero. For each atom-projected density of states, the units correspond to 3 states/eV, while the transmission results are displayed between 0 and 1.

where $T(E,V)$ is the total (spin-up plus spin-down) transmission coefficient represented in Figure S3, $f(E)$ is the Fermi function at a given temperature, and μ_L and μ_R are the chemical potentials of the left and right leads, respectively. Due to the difference between the two Fermi functions, the integration range is limited to the energy window between μ_L and μ_R (with a broadening related to the temperature). When a bias V is applied to the molecular junction, μ_L (resp. μ_R) is shifted up (resp. down) by $eV/2$:

$$\mu_{L/R} = \mu \pm eV/2, \tag{3}$$

as illustrated in Fig. 1 of Ref. 2. Hence, the integration range in Eq. (2) increases as illustrated by the dashed white lines in Figure S3.

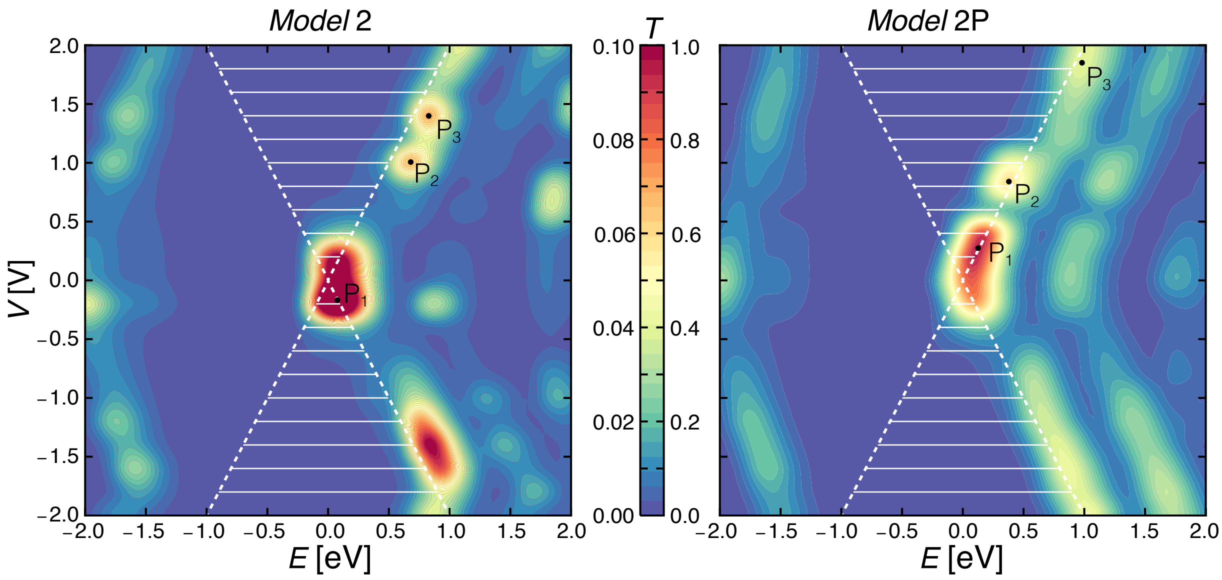

Figure S3: Contour maps of the total transmission T as a function of the energy E and the applied bias voltage V for *Models* 2 and 2P. In contrast with Fig. 3 of the main text, a different scale has been chosen for the two *Models* in order to highlight the maxima. The Fermi energy is set to zero on the energy axis. The white dotted lines indicate the integration range in Eq. (2) between the chemical potentials of the left and right leads as a function of the bias voltage V. The white solid lines show the energy windows that have been explicitly considered for calculating the $I(V)$ characteristics in Fig. 2 of the main text (*i.e.* by steps of 0.2 V).

The $I(V)$ characteristics in Fig. 2 of the main text can be easily understood by inspect-

ing Figure S3. For *Model* 2 (representative for *Models* 1-6), the peak P_1 in $T(E, V)$ around (0.1 eV, -0.2 V) is responsible for the peaks at $V = \pm 0.2$ V in the $I(V)$ curve. The peaks P_2 around (0.7 eV, 1.0 V) and P_3 around (0.9 eV, 1.4 V) which lie slightly outside the integration range, translate into a plateau in the current. So, the NDR is related to the valley between the peaks P_1 and P_2. For the *Model* 2P, the peak P_1 around (0.3 eV, 0.3 V) is quite extended and gives rise to the peaks at $V = -0.2$ and 0.4 V (due to its asymmetric shape). On the positive bias side, the current peak is followed by a dip at $\sim$0.6 V, which induces a NDR, due to the valley between P_1 and P_2 around (0.5 eV, 0.9 V). In contrast with *Model* 2, the separation between the peaks P_2 and P_3 around (1.0 eV, 1.9 V) is pronounced and induces a new dip in the current, and hence a NDR behaviour.

2.2 Single and packed ruthenium-terpyridine molecules between two Au(111) electrodes: Polarization

Insight into the behaviour of the molecular junctions can be obtained by re-plotting the $I(V)$ curves as polarization traces $P(V)$, as shown in Figure S4. We observe that for *Models* 1 - 3 the geometry of the devices is not strongly correlated with the polarization, which does not change if the devices are probed at negative or positive biases. The calculated $P(V)$ for the packed system could be understood as arising from the lowest unoccupied molecular orbital (LUMO) given by the superposition of the two LUMOs of the constituent molecules (see below).

2.3 Single and packed ruthenium-terpyridine molecules between two Au(111) electrodes: Magnetization

The calculated magnetization curves $m(V)$ are displayed in Figure S5 for all investigated cases. The shape of the magnetization peak around zero bias is indicative for a peculiar distribution of the magnetization density across the molecular constituents of the devices.

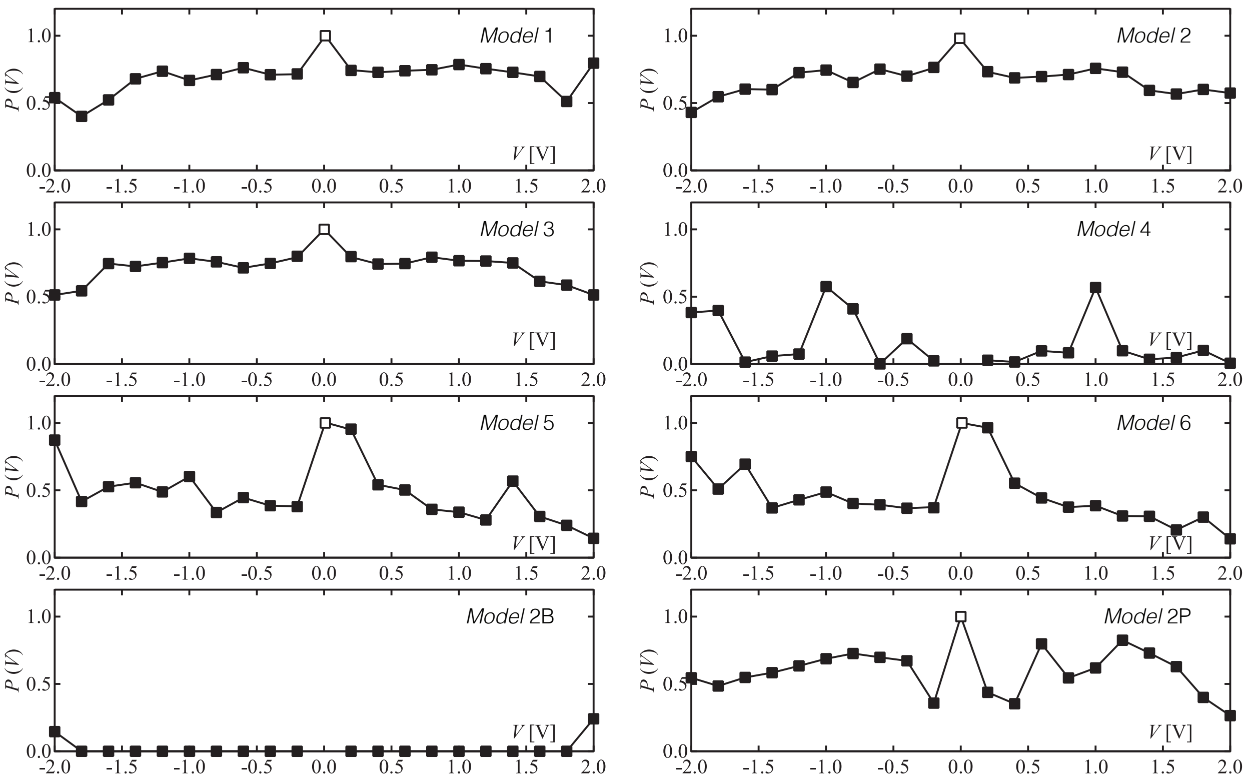

Figure S4: Calculated polarization as a function of the applied bias V for all studied *Models* 1 - 6, 2B, and 2P. At non-zero bias, the polarization is obtained as $P(V) = |I_{\uparrow}(V) - I_{\downarrow}(V)|/|I_{\uparrow}(V) + I_{\downarrow}(V)|$. At zero bias, it is computed differently as $P(0) = |T_{\uparrow}(E_F) - T_{\downarrow}(E_F)|/|T_{\uparrow}(E_F) + T_{\downarrow}(E_F)|$, except for *Models* 4 and 2B whose total transmission $T(E_F)$=0. Hence, it is reported with a different symbol (an open rather than a filled square).

Indeed, the close interrelation between the values of the magnetic moment and the localisation of the spin-up and spin-down densities on the terpyridine ligands is supported by the spatial mapping of the magnetization density at zero and low bias voltages (see main text and the following Subsection).

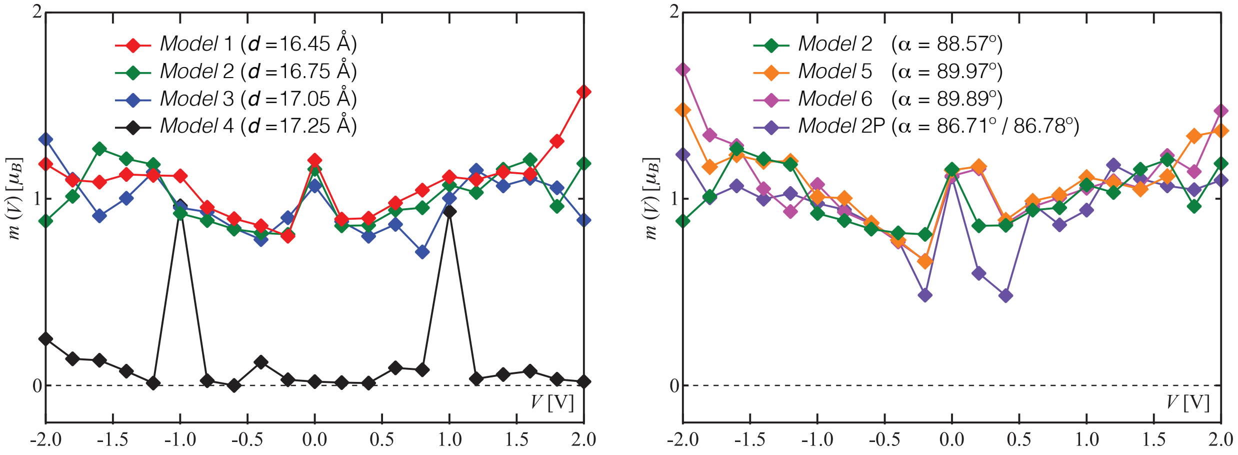

Figure S5: Calculated magnetization m (given in μ_B) as function of the applied bias V for all studied cases. The right panel shows a comparison of the *Models* 1 - 4 whose geometry differ only by the inter-electrode distance. The left panel displays a comparison of the *Models* 2, 5, 6, and 2P that have a common inter-electrode distance $d = 16.75$ Å, but differ by the dihedral angle α between the two rigid terpyridine ligands and the molecular packing.

In order to analyze the sudden change in the magnetization around V=±1 V for *Model* 4, we report the PDOS results for different bias voltages (V = 0.8, 1.0, and 1.2 V) in Figure S6. While the spin-up and spin-down PDOS almost compensate each other (see green curves for their difference) at V = 0.8 and 1.2 V, their difference becomes quite important at V = 1.0 V especially for the C, N, and Ru atoms. Furthermore, we note that the HOMO (which has a large PDOS on C atoms) is located almost exactly at the Fermi level for V = 1.0 V, while it falls slightly below for V = 0.8 and 1.2 V. As a consequence, when integrating the PDOS below the Fermi level for each atomic species, the difference between the resulting spin-up and spin-down populations is extremely small for all species at V = 0.8 and 1.2 V but it is clearly non-zero for C atoms (and N atoms to a lesser extent) at V = 1.0 V. This is perfectly in line with what is observed in Figure 4 for the magnetization

density.

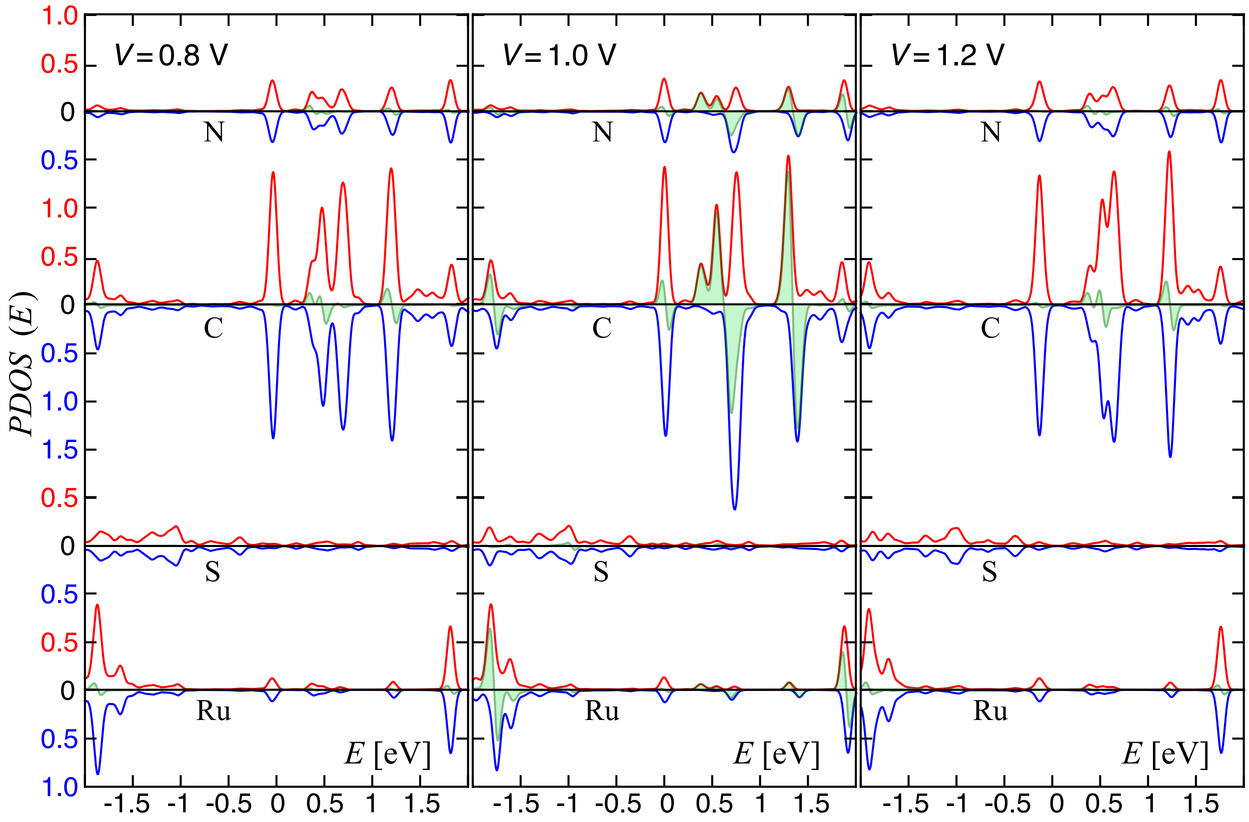

Figure S6: Atom-projected density of states (PDOS) for *Model* 4 at different bias voltages (V = 0.8, 1.0, and 1.2 V). We use red curves for spin-up, blue curves for spin-down data, and green curves for their difference. The Fermi level was set to zero. For each atom-projected density of states, the units correspond to 3 states/eV, while the transmission results are displayed between 0 and 1.

2.4 The space-resolved spin density

The space-resolved spin density for the *Model* 2P is represented at various bias voltages (V = 0.0, 0.2, 1.0 and 2.0 V) in Figure S7 . The overall behaviour of the packed ruthenium-terpyridine molecules linked between two Au(111) electrodes is imposed by the Molecule 2, which is tightly linked to the bottom electrode. At large bias configuration, the magnetisation density is essentially localised on the terpyridines linked to the bottom electrode.

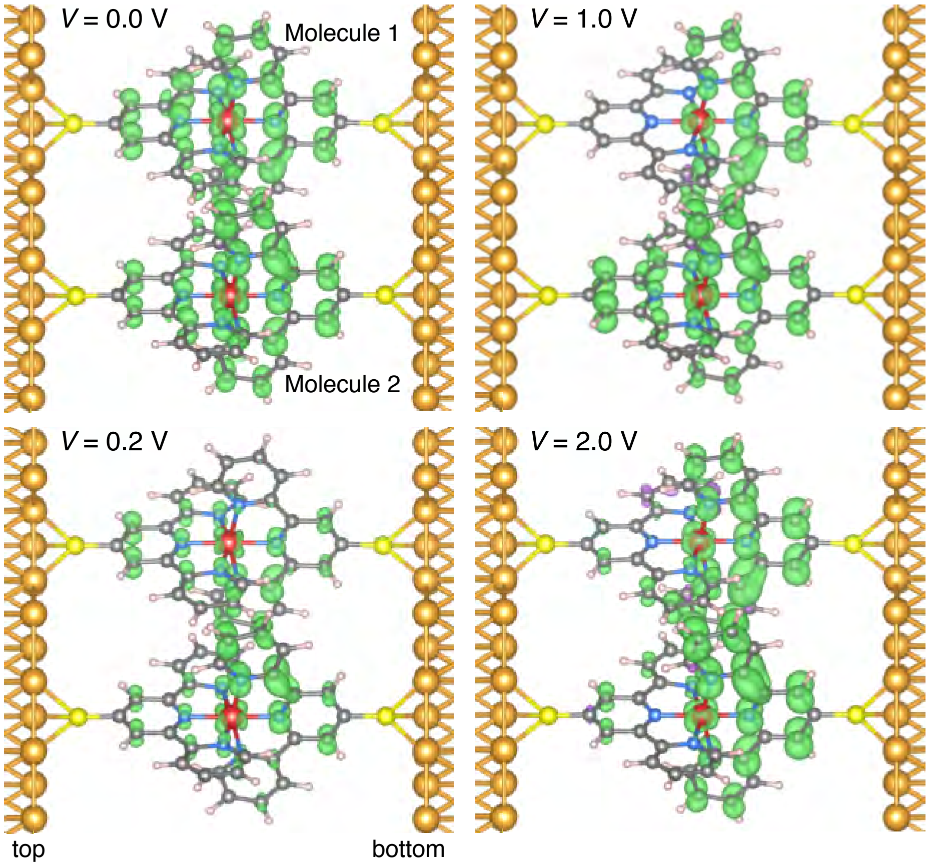

Figure S7: Magnetization density for the *Model* 2P at various bias voltages (V = 0.0, 0.2, 1.0 and 2.0 V). The atoms are represented adopting the same color scheme as in Figure 1 (main text). The isosurfaces in green and violet correspond to a magnetization density of $\pm 2 \times 10^{-3}\ \mu_B/\mathrm{Bohr}^3$.

3 Computed electronic properties of the isolated molecule

Figure S8 shows the energy dependence of the density of states (DOS) for the isolated molecule as calculated using the PBE exchange-correlation functional. The contour plot representation of the highest occupied molecular orbital (HOMO) and LUMO is given in the inset to Figure S8. The isolated radical has a small HOMO-LUMO gap of about 0.5 eV and both HOMO and LUMO orbitals are twofold degenerate. Each of the twofold degenerate LUMO orbitals is mainly located on a different terpyridine unit. No magnetic moment is present for the isolated radical.

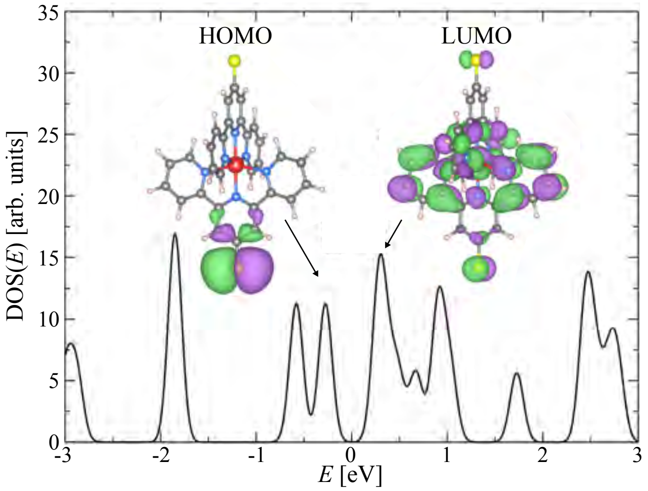

Figure S8: Energy dependence of the density of states for the free radical. The vacuum energy was set to zero. Contour plots for the HOMO and LUMO orbitals are drawn in the inset. The isosurfaces in green and violet correspond to $\pm 2 \times 10^{-3}$ e/Bohr^3. Note that, due to the symmetry of the molecule, the HOMO-1 (resp. LUMO+1) is degenerate with the HOMO (resp. LUMO). The corresponding contour plots (not reported here for sake of clarity) are very similar to the HOMO and LUMO shown in the figure, but spatially localized on the other terpyridine unit.

Since the conventional DFT exchange-correlation functionals (such as PBE) tend to underestimate the difference between the HOMO and LUMO energies (E_g), we have also performed the calculations using hybrid functionals (PBE0 and B3LYP) and computed many-body corrections (using both the one-shot G_0W_0 method and the self-consistent

evGW approach with self-consistency on the eigenvalues) for the isolated molecule. The calculations with the hybrid functionals where performed using the NWCHEM code, while the many-body corrections were obtained with the FIESTA code. The many-body corrections were obtained starting from the PBE0 eigenvalues and wavefunctions. For both DFT and many-body calculations, changing the basis set from cc-pVTZ to aug-pVTZ affected the value of E_g by less than 0.1 eV. The results are presented in Table S2.

Table S2: Difference between the HOMO and LUMO energies (E_g) as calculated using DFT with the PBE, PBE0, and B3LYP exchange-correlation functionals and including many-body corrections (using both the one-shot G_0W_0 method and the self-consistent evGW approach with self-consistency on the eigenvalues).

Method	PBE	PBE0	B3LYP	G_0W_0	evGW
E_g	0.5	0.9	0.7	2.3	2.5

These values can be used to correct the eigenvalues for the devices using the the DFT+Σ method.[3–5] This requires to also take into account an "image charge" term, accounting for the polarization energy associated with static nonlocal correlations between the electrons on the molecule and in the metal electrodes that closes the gap of the molecule upon absorption. Typically, this will reduce the difference between the HOMO and LUMO energies of the isolated molecule by 35-45%.[6] As a result, we expect a separation of ∼1.5 eV for the system considered here.

References

(1) See, for example, for a recent review, *Architecture and design of molecule logic gates and atom circuits.* N. Lorente and C. Joachim eds. (Springer, Berlin Heidelberg, 2013).

(2) Rocha, A. R. *et al.* Spin and molecular electronics in atomically generated orbital landscapes. *Phys. Rev. B* **73**, 085414 (2006).

(3) Quek, S. Y., Venkataraman, L., Choi, H. J., Louie, S. G., Hybertsen, M. S. & Neaton, J. B. Amine-gold linked single-molecule circuits: Experiment and theory. *Nano Letters* **7**, 3477 (2007).

(4) Quek, S. Y., Choi, H. J., Louie, S. G. & Neaton, J. B. Length dependence of conductance in aromatic single-molecule junctions. *Nano Letters* **9**, 3949 (2009).

(5) Darancet, P., Widawsky, J. R., Choi, H. J., Venkataraman, L. & Neaton, J. B. Quantitative current–voltage characteristics in molecular junctions from first principles. *Nano Letters* **12**, 6250 (2012).

(6) Neaton, J. B., Hybertsen, M. S. & Louie, S. G. Renormalization of molecular electronic levels at metal-molecule interfaces. *Physical Review Letters* **97**, 216405 (2006).
